# Temporal Assessment of Gadolinium Deposition and T1 Signal Intensity Changes in Rat Kidney with Single and Multiple Doses of Injection: An Experimental Study

**DOI:** 10.5152/eurasianjmed.2024.23155

**Published:** 2024-02-01

**Authors:** Fatih Alper, Adem Karaman, Ahmet Yalçın, Alptug Atila, Busra Dincer, Busra Diyarbakir Sirinoglu, Serhat Kaya, Taha Tavaci

**Affiliations:** 1 Department of Radiology, Atatürk University Faculty of Medicine, Erzurum, Turkey; 2 Department of Analytical Chemistry, Atatürk University Faculty of Pharmacy, Erzurum, Turkey; 3 Department of Pharmacology, Erzincan Binali Yıldırım University Faculty of Pharmacy, Erzincan, Turkey; 4 Department of Pharmacology, Atatürk University Faculty of Medicine, Erzurum, Turkey

**Keywords:** Gadolinium deposition, rat kidney, T1 image intensity

## Abstract

**Background:**

Gadolinium deposition in biological tissues was first reported in patients with renal failure. We aimed to investigate gadolinium deposition in the rat kidney after exposure to single and multiple doses of gadolinium and evaluate deposition for 1- and 3-month periods. We also aimed to determine any correlation between the amount of deposition and T1-weighted image intensity.

**Methods:**

Seventy rats (5 animals per group) were included in the sample, and 9 groups received a single dose (0.3, 0.6, and 1.2 mmol/kg) of gadolinium, and 1 group for each dose was sacrificed at the end of the first day, week, and month. Four groups received weekly doses (0.3 and 0.6 mmol/kg) and were sacrificed at the end of 6 and 12 weeks. Measurement of T1 intensities was carried out with postinjection images before sacrifice, and deposition was determined using inductively coupled plasma mass spectrometry.

**Results:**

The number of injections was associated with increased gadolinium deposition (*P* < .001) in the kidney. After the weekly injections, the deposited gadolinium levels did not significantly differ between the low and medium doses at the end of the sixth week (*P* = .067). There was no agreement between the observers regarding the measurement of T1 signal intensity in both single-dose and multidose experiments (*P* = .263 and *P* = .307, respectively).

**Conclusion:**

Deposition was dose dependent in the postinjection stage in contrast to the late stage in which deposition was not associated with dose or number of injections until the 12th week. T1 signal intensity measurement is unreliable for assessing deposition in the rat kidney.

**Main Points**

- After single-dose injection, gadolinium deposition in rat kidneys was robust and dose dependent until the fourth week.
- After multidose injection, the deposition was not associated with the dose and number of injections.
- Measurement of T1 signal intensity was not a reliable assessment method for the evaluation of gadolinium deposition in rat kidneys.

## Introduction

Gadolinium (Gd) deposition was first described in the early 2000s as a form of a disease called nephrogenic systemic fibrosis (NSF)/dermatopathy.^1-3^ In initial studies, this deposition was found to be associated with the fibrosis of skin tissue in patients with renal failure, whereas Gd was identified as the culprit in later studies.^4-6^ A decade after the discussion of the relationship between Gd and NSF, researchers have begun to investigate Gd deposition in tissues in patients with normal renal function by utilizing the T1-weighted image signal and inductively coupled plasma mass spectrometry (ICP-MS).^7,8^ In these studies, increased signal intensity on noncontrast T1 images was used as an indirect indicator of Gd deposition in patients who had previously undergone MR imaging with Gd-containing contrast agents. On the other hand, ICP-MS was used for the quantification of Gd deposition in tissues. However, the latter technique requires tissue sampling for analysis.

After the injection of the agent that contains Gd, virtually all the excretion occurs via the kidneys, except for liver-specific agents.^9^ Several studies in the literature have focused on the deposition of Gd in the brain, bone, and skin,^8,10-15^ but there are fewer papers aimed at investigating the retention of this element in the kidney,^16,17^ and to our knowledge, no study has evaluated the renal deposition of Gd according to dose and number of injections in a given time.

Since Gd deposition in biological tissues was first reported in patients with renal failure, we consider that the detection of renal retention of Gd and its relationship with the number of injections and the dose might give a clue to understanding the mechanism of retention in the human body. In this study, we investigated whether the renal deposition of Gd differed between single and multiple injections of various doses. The identification of a renal excretion pattern with different doses of administration and observation of deposition over a long term can guide the development of new treatment strategies for Gd deposition or restriction of use. We also used magnetic resonance imaging (MRI) signal intensity to compare the degree of deposition, similar to previous studies, show the reliability of MRI, and identify the effect of deposition on imaging.

In this experimental animal study, we aimed to investigate Gd deposition in the rat kidney after exposure to single and multiple doses of Gd and evaluate its retention levels for 1- and 3-month periods. We also aimed to determine any correlation between the amount of deposition and the T1-weighted intensity of the kidneys based on images acquired at different time intervals.

## Material and Methods

This study was performed at the Experimental Animal Laboratory of Medicinal and Experimental Application and Research Center of Atatürk University. All procedures followed the national guidelines for the use and care of laboratory animals and were approved by the local animal care committee of the university (protocol number: 2016-160, date: 06.12.2016).

### Animals and Chemicals

In the experiments, 70 Wistar albino male rats weighing 250-280 g obtained from the Experimental Animal Laboratory of Medicinal and Experimental Application and Research Center were used. The animals were housed under controlled conditions at 22 ± 1°C and 12-hour light–12-hour darkness cycles in standard plastic cages. Rat food pellets and water were provided ad libitum. Gadodiamide was used as a contrast agent (Omniscan, Opakim Pharma, Istanbul, Turkey), and 50 mg/mL ketamine (Ketalar, Pfizer, Istanbul, Turkey) and 100 mg/mL xylazine (XYLASED, Bioveta, Ankara, Turkey) were used as dissociative anesthetics.

### Experimental Design

**Figure 1.**
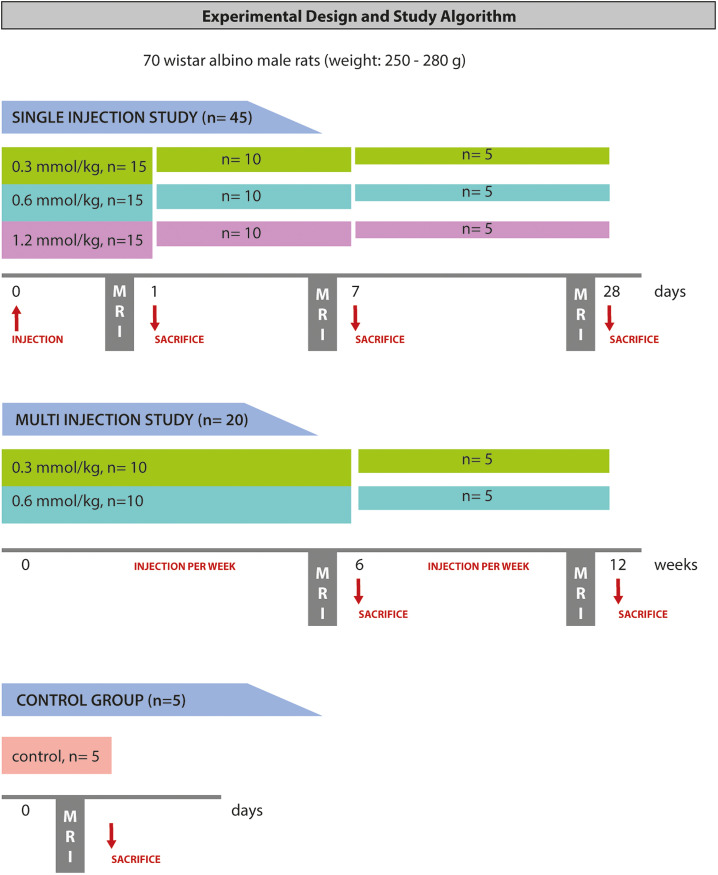
Study design and flow algorithm. MRI, magnetic resonance imaging.

This study was designed in 2 stages as single- and multidose experiments. In both stages, a linear contrast agent (gadodiamide) was administered intravenously through the tail vein at a dose of 0.3, 0.6, and 1.2 mmol/kg. A dose of 0.6 mmol/kg corresponds to 0.1 mmol/kg in humans after adjusting for body surface area according to the U.S. Food and Drug Administration guidelines.^18^ This dose was halved (lower dose) and doubled (higher dose) to assess dose-dependent retention. Group allocation and study algorithm are summarized in Figure 1. The animals were sacrificed 3 hours after MRI examinations in all groups.

### Imaging Technique and Assessment

All MRI examinations were performed with a 3 Tesla scanner (Skyra, Siemens Healthcare, Erlangen, Germany). The anesthetized rats were imaged using a dedicated 16-channel knee coil, and T1-weighted images were acquired using the multi-shot turbo spin-echo sequence with the following parameters: repetition time msec/echo time msec, 700/12 msec; average, 1; slice thickness, 1 mm; interslice gap, 0; voxel size, 0.8 × 0.8 × 1 mm; field of view, 200 mm; and matrix, 248 × 256.

**Figure 2.**
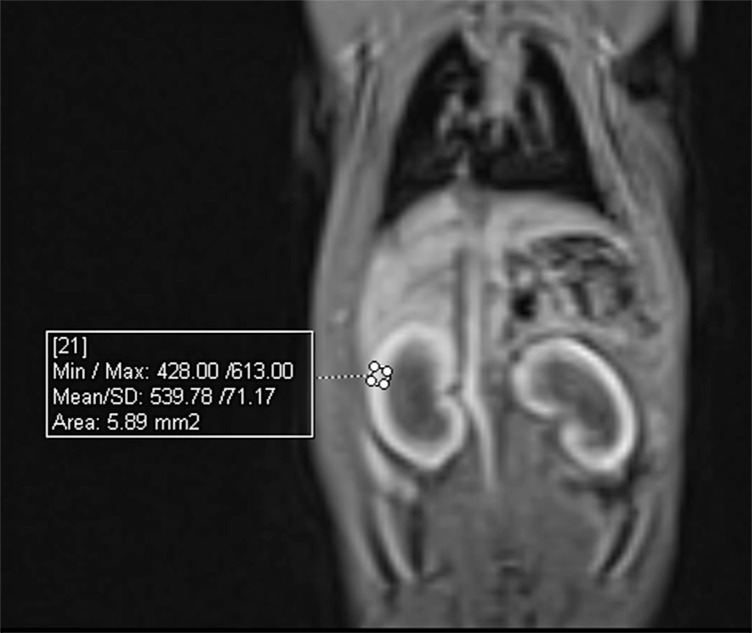
Coronal T1-weighted, contrast-enhanced image showing the regions of interest method for the measurement of renal parenchymal signal intensity.

All the images were transferred to an offline workstation (Syngo via Siemens, Erlangen, Germany). The intensity of the kidneys was measured using circular regions of interest (ROI) on the coronal T1-weighted images by 2 radiologists who were blinded to data regarding the doses and injection times (Figure 2). A total of 6 ROIs were placed in the upper, middle, and lower poles (2 for each) of the kidneys. The mean intensity of the right and left kidneys was determined by calculating the mean values of the ROI. The mean kidney intensity for both observers was determined based on the arithmetic mean of the left and right kidneys.

### Determination of Gadolinium Levels

The samples were prepared as 0.5 grams maximum with the addition of 8 mL HNO3 and 2 mL H_2_O_2_ solution. After microwave decomposition, the samples were filtered using a 0.45 µm syringe filter and read by the device without a second dilution. Each reading indicated the average of 3 parallel readings. The ICP-MS Agilent 7800 series (Agilent Technologies, Tokyo Japan) was used to determine the concentration of the Gd element in the solution. Mass Hunter 4.2 Workstation (Software 7800 ICP-MS Top C.01.02) was used to calculate the measurements. The element concentrations were automatically calculated in parts per billion (ppb) by the software according to the formula given below. The ppb concentration values were converted to mg/kg and recorded for each sample. A detailed description of the analysis is given in Appendix 1.

Dilution factor = (final weight or volume/sample quantity or volume) × dilution coefficient

### Statistical Analysis

The normality of the data was assessed using the Shapiro–Wilk test. One-way analysis of variance was used for the comparison of more than 2 groups, whereas the *t*-test was used for the comparison of 2 groups. The Pearson correlation was used for the assessment of a positive or negative correlation between Gd deposition and T1-weighted image intensity. A 2-way intraclass correlation coefficient (ICC) was used to assess the agreement between the observers. A 2-tailed *P* < .05 was considered statistically significant. All statistical analyses were conducted using the R statistical package (R Studio, Vienna, Austria).

## Results

### Single-Dose Experiment

**Figure 3.**
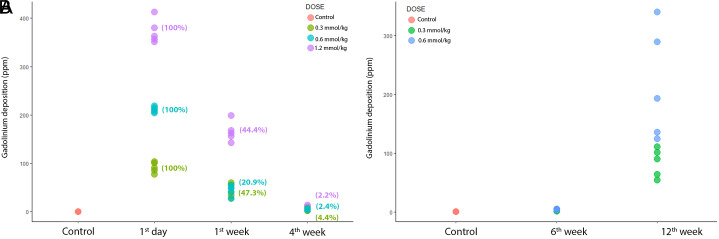
Scatter diagram showing the levels of gadolinium deposition according to time points and different doses in the single-dose (A) and multidose (B) experiments (see text for *P*-values). Numbers in parentheses show the percentages of changes in deposition in each group.

The mean Gd deposition levels in the kidney tissue were 225 ± 120 mg/kg, 84 ± 61 mg/kg, and 6 ± 3 mg/kg at the end of the first day, first week, and fourth week, respectively. For all the time points, the levels of Gd deposition were significantly different from each other (first day versus first week, *P* < .001; first week versus fourth week, *P* < .001; and first week versus fourth week, *P* = .022) according to the Tukey test of the multiple comparisons of means (Figure 3A).

At the end of the first day, the mean Gd deposition levels in the kidney tissue were 91 ± 10 mg/kg, 211 ± 5 mg/kg, and 372 ± 25 mg/kg for the low, medium, and high doses of Gd, respectively. For all the dose groups, the levels of Gd deposition were different from each other (*P* < .001 for all) (Figure 3A). At the end of the first week, the mean Gd deposition levels were 43 ± 14 mg/kg, 44 ± 10 mg/kg, and 165 ± 20 mg/kg for the injections of low, medium, and high doses of Gd, respectively. The high-dose group had significantly higher Gd deposition in the tissue compared to the medium and low doses (*P* < .001 for both), but there was no statistically significant difference between the medium and low doses (*P* = .996) (Figure 3A). At the end of the fourth week, the mean Gd deposition levels were 4 ± 2 mg/kg, 5 ± 1 mg/kg, and 8 ± 4 mg/kg for the injections of the low, medium, and high doses of Gd, respectively. There was no statistically significant difference between the dose groups about the Gd deposition levels (*P* = .127) (Figure 3A).

**Figure 4.**
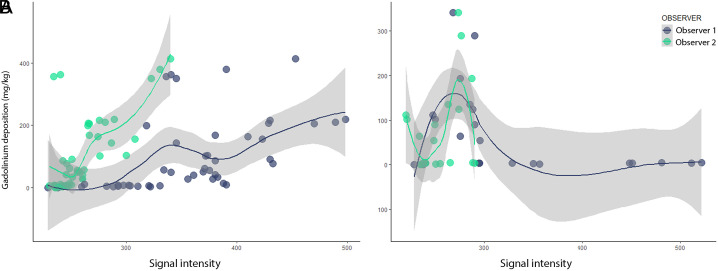
Scatter diagrams with trend lines showing the relationship between measured signal intensities and the amount of gadolinium deposition in the single-dose (A) and multidose (B) experiments (see text for *P*-values).

In the single-dose experiment, the amount of deposited Gd in the tissue was well correlated with the mean intensity of the kidneys in T1-weighted images for both observer 1 and observer 2 (*r* = 0.513, *P* < .001 and *r* = 0.689, *P* < .001, respectively); however, the agreement between the 2 observers was not significant (ICC = 0.227, 95% CI = −0.230-0.603, *P* = .263) (Figure 4A).

### Multidose Experiment

At the end of the sixth week and 12th week after weekly administration of Gd, mean Gd levels in the kidney tissue were 3.7 ± 1.0 mg/kg and 150.3 ± 95.8 mg/kg, respectively and the latter was significantly higher than the former (*P* < .001) (Figure 3B).

At the end of the sixth week, mean Gd deposition levels were 3.3 ± 0.9 mg/kg and 4.3 ± 0.7 mg/kg for low and medium doses, respectively. There was no statistical significance between dose groups (*P* = .067) (Figure 3B). At the end of the 12th week, mean Gd deposition levels were 84.1 ± 24.4 mg/kg and 216.5 ± 95.3 mg/kg for the low and medium doses, respectively. The medium-dose group had significantly higher Gd deposition levels (*P* = .033). There was also no significant difference between low and medium doses belonging to the sixth week of the multidose experiment and the fourth week of the single-dose experiment (*P* = .273 and .091, respectively).

In the multidose experiment, intensity calculated from the T1-weighted images was negatively well correlated with Gd deposition for observer 1 (*r* = −0.544, *P* = .013), but there was no such correlation for observer 2 (*r* = 0.322, *P* = .166). There was also no agreement between the 2 observers (ICC = 0.139, 95% CI = −0.390-0.562, *P* = .307) (Figure 4B).

## Discussion

The results of our study revealed that after a single-dose injection, Gd retention was reduced rapidly and reached the lowest mass fraction at the end of the fourth week when the amount of retention was not significant for any of the doses. Before that time, higher doses of Gd injections were associated with higher mass fractions of Gd deposited in the kidney tissue. Signal intensity in the MRI images was correlated with the amount of Gd fraction deposited in the tissue for both observers, but there was no interobserver agreement. After the weekly injection of multiple doses for 12 weeks, the measured mass fraction of deposited Gd did not significantly differ between the low and medium doses at the end of the sixth week, and it was similar to the result obtained at the end of the fourth week with the use of a single dose. At the end of the 12th week, retention was remarkable and dose dependent. Signal intensity measurements were noncorrelated with the amount of retention and inconsistent between the 2 observers.

In this study, renal Gd deposition after a single dose in normal kidneys was dose dependent in the immediate postinjection period. Over time, retention regressed to a minimum amount without indifference in the low-, medium-, and high-dose groups. This suggests a clearance mechanism that is capable of removing a major amount of Gd from the circulation. In earlier stages, this mechanism was restricted by the higher doses of Gd; however, the amount of retention was not affected by the dose of Gd injected in the later stages. The multidose experiment showed that different doses with multi-injections did not alter this mechanism, and the result was similar to the last week of the single-dose experiment. After more than 10 injections, the kidneys were unable to remove Gd, and the increased retention was, again, dose dependent.

The retention of Gd in normal functioning kidneys was assessed previously, and the authors found that the amount of Gd retention in the rat kidney depended on the type of Gd chelate given after 20 injections.^16^ In that study, with the injection of gadodiamide, the amount of Gd retention in the kidney was found to be higher than in any other organ, including the brain, liver, and spleen.^16^ This indicates that, in addition to the excretory role, the kidney is the main site of Gd deposition. In another study, the authors found that macrocyclic agents could also be deposited primarily in the kidney after a 28-day recovery period following multiple injections of Gd.^17^ In various animal and human studies, the deposition of Gd (especially in the brain) is found to be associated with the dose, type of Gd chelate, and the number of injections ^17,19-21^ although there is no study in the literature that has assessed renal deposition. Furthermore, studies conducted on Gd deposition lack multitime evaluation and perform such assessments only once. Our study revealed that the deposition process in a dynamic event and deposition-time graph was not linear. The toxic effects of Gd on the rat kidney were previously reported in the literature, and increased serum urea, calcium, enlarged Bowman capsules, and shrunk distal and proximal tubules were found to be associated with Gd deposition.^22^ In that study, the authors also found histopathologic changes associated with Gd administration at a dose of 2 mmol/kg, which is above the standard dose.^22^ The abovementioned renal damage can also explain our findings after 12 weeks of multi-injections of Gd.

Another method to assess Gd deposition in tissues, mainly brain tissue, is the measurement of T1 signal intensity changes in non-contrast-enhanced brain images. This was first described in 2015, and the authors found that T1 signal intensity was correlated with the amount of Gd deposited in the brain tissue, which was analyzed in the postmortem period.^8^ Similar to retention studies, several studies linked T1 signal alterations in non-contrast-enhanced images to the type of Gd chelate and number of injections.^11,14,23^ However, later studies reported opposite results.^24-26^ Since there is no other study that evaluated T1 signal intensity in the kidneys after the single and serial administration of Gd, we were not able to compare our results, but they indicated that T1 signal intensity was correlated with the amount of Gd retention in the kidney only during the early postinjection period. At later stages, when lower levels of Gd deposition were present, no such correlation was observed. Another important finding is that even in the presence of such a correlation in the early period, there was no agreement between the 2 observers. This suggests that measuring the T1 signal is an unreliable method for the indirect detection of Gd retention.

Based on the findings obtained from our experimental study, we concluded that Gd retention in the kidney tissue was directly related to the excretion function of the kidneys in the early stages. The excretion of Gd was dose dependent, whereas its retention in the kidney was independent of the dose, as indicated by our fourth-week data. In the long term with repeated injections, the findings were similar to those at the end of the first week of the single-injection experiment, except for the terminal stage, in which renal functions deteriorated and renal retention was significantly increased. We also concluded that the effect of Gd on the reduced relaxation time of water molecules could not be used as a marker for the detection of the amount of Gd retention.

We also considered some of the clinical aspects of this study. In the literature, certain methods are recommended to decrease Gd deposition in tissues.^27-29^ Since our findings revealed that the most notable and dose-dependent deposition occurred in the kidney in the early stages of postinjection, we think that the prevention of deposition should be aimed at this stage. Deposition in the kidney tissue was reduced independently from time until a point at which the kidneys were no longer able to compensate for this deposition. We conclude that kidneys have a certain limit for the compensation of Gd deposition, and when this limit is exceeded, retention occurs rapidly. Results of the multidose experiment imply that repetitive injections might compel the kidneys to use ability to remove Gd from the renal tissues.

Our study has certain limitations. First, we did not obtain histopathological specimens of the kidneys to show the effect mechanism of Gd deposition and pathologic tissue alterations. Second, we only used a linear agent; therefore, we do not know whether the retention pattern would have been similar if we had used macrocyclic agents. Third, we conducted the experiments on an animal model, and the same study algorithm should be followed in studies on human subjects to confirm our findings. The length of stages that was determined in our study for rats would differ from humans, considering that 1 day of rat life corresponds to 34.8 days of human life.^30^ Finally, our sample size was relatively small, and our results might need validation from larger studies.

We found that at the early postinjection stage, the deposition of Gd in the kidneys was remarkable and dose dependent in contrast to the late stage in which low levels of final deposition were not associated with the dose or number of injections until the 12th week. We also found that T1 signal intensity was not a reliable tool for assessing Gd deposition in the rat kidney.
